# Hydroxyapatite-Fluoride Toothpastes on Caries Activity: A Triple-Blind Randomized Clinical Trial

**DOI:** 10.1016/j.identj.2024.09.037

**Published:** 2025-02-18

**Authors:** Fabio Cocco, Claudia Salerno, Richard Johannes Wierichs, Thomas Gerhard Wolf, Antonella Arghittu, Maria Grazia Cagetti, Guglielmo Campus

**Affiliations:** a Department of Surgery, Microsurgery and Medicine Sciences, School of Dentistry, University of Sassari, Sassari, Italy; b Department of Biomedical, Surgical and Dental Sciences, University of Milan, Milan, Italy; c Department of Restorative, Preventive and Pediatric Dentistry, University of Bern, Bern, Switzerland; d Graduate School for Health Sciences, University of Bern, Switzerland; e Department of Periodontology and Operative Dentistry, University Medical Center of the Johannes Gutenberg-University Mainz, Mainz, Germany; f Direzione Igiene e Controllo delle Infezioni Ospedaliere, University Hospital of Sassari, Sassari, Italy; g Department of Cariology, Saveetha Dental College and Hospitals, SIMATS, Chennai, India; h Department of Cariology, Institute of Odontology, Sahlgrenska Academin, University of Gothenburg, Gothenburg, Italy

**Keywords:** Caries management, Children, Fluoride, Nano-hydroxyapatite, RCT, Toothpaste

## Abstract

The study aimed to evaluate the remineralizing effect of hydroxyapatite and fluoride containing toothpastes (HAF's) on active caries lesions compared to a fluoridated standard toothpaste in pre/schoolchildren. A total of 610 children (4-5 and 6-7 years old) were enrolled. Four toothpastes, 2 containing fluoride-substituted hydroxyapatite (HAF) (1000 and 1450 ppmF) and magnesium-, strontium-, carbonate-substituted hydroxyapatite, in a chitosan matrix and 2 Mono fluoridated toothpastes (NaMFP) (1000 and 1450 ppmF), were randomly administered for 24 months. The children were instructed to brush for 2 minutes 3 times/day. Caries activities, by clinical surface features, were recorded at baseline and 12- and 24-month follow-ups. A per-protocol analysis was adopted, thus excluding children lost to follow-up. Overall, 518 children completed the trial. Baseline comparisons revealed no significant differences in primary teeth caries rates between HAF and NaMFP groups, both for enamel and dentinal lesions. By the end of the study, the HAF group exhibited a statistically significant reduction in enamel lesions compared to the NaMFP group (*P* < .01). Of the 40 partially active lesions at baseline in the HAF group, 13 were inactive at the 2-year follow-up. Of the active lesions in the HAF group (n = 78) at baseline, nearly 3-quarters (n = 58) were inactive at the follow-up. The difference between the 2 groups (HAF *vs* NaMFP) in terms of change of status in primary dentition (active at baseline and inactive at follow-up) was statistically significant (*P* = .04). Regarding dentinal lesions, both groups presented similar percentages of inactive lesions that were filled at the end of follow-up (*P* = .08). However, the HAF group demonstrated a higher number of inactivated lesions compared to the NaMFP group. The toothpaste containing biomimetic hydroxyapatite and fluoride may be better for children with active caries lesion in primary dentition.

## Introduction

Although preventable, dental caries remains 1 of the most prevalent noncommunicable chronic diseases among children worldwide and 1 of the most common unmet healthcare needs among disadvantaged children.^1^, ^2^, ^3^

Early childhood caries (ECC) is the early onset of caries in young children with the presence of 1 or more decayed (noncavitated or cavitated lesions), missing (due to caries), or filled surfaces in any primary tooth of a child under the age of 6.^4^ ECC is a major public health issue worldwide, impacting the dental health and well-being of young children. ECC is often treated using a multidisciplinary approach and different preventive strategies are needed and recommended to control caries risk, mainly based on dietary changes or enhancing host resistance, such as using fluoride-containing toothpaste twice a day.^5^ Fluoride-containing toothpastes are still the most effective and affordable means of preventing caries, primarily when other community-level preventive strategies are not implemented.^1^^,^^6^ Fluoride, including that contained in toothpaste, has the most consistent benefit in preventing the development of caries lesions, re-mineralizing initial noncavitated lesions,^7^ and arresting the caries process even when lesions are already cavitated.^8^ However, non-cavitated lesions may differ considerably in terms of the risk of progression.^4^^,^^9^ Caries lesion activity assessment differentiates active from inactive lesions to provide appropriate treatment planning for active lesions, focusing on arresting progression.^4^ Several systems have defined the process to assess the activity of the lesion based on tactile and visual examinations.^8^^,^^10^^,^^11^

While most of the literature on fluoridated toothpaste has demonstrated its ability to prevent the development of caries,^12^^,^^13^ there is low to moderate scientific evidence that fluoridated toothpaste can reverse caries activity by achieving a remineralizing effect of the lesion.^14^

Recently, there has been a concerted effort to find effective nonfluoride caries inhibitors. However, as products also strive to improve overall oral health by reducing the risk of gingivitis, reducing dentinal sensitivity, preventing tooth mineral loss and improving tooth appearance, the active ingredient must be versatile and provide more than 1 benefit.^15^, ^16^, ^17^ The most promising active ingredients in toothpastes to achieve all these goals are calcium phosphate molecules.^15^ Hydroxyapatite, a bioactive and biocompatible material with broad applications in both medicine (*eg*, bone substitute) and dentistry, is currently used in nano-crystalline forms in toothpaste and mouth rinse in varying concentrations for caries prevention, hard tissue remineralization and dentin hypersensitivity. Hydroxyapatite, a natural component of tooth enamel, has the ability to remineralize and desensitize exposed dentin surfaces by forming a protective layer that blocks dentinal tubules, reducing the transmission of external stimuli that cause sensitivity.^16^

One of the new technologies proposed includes synthetic hydroxyapatite HA [Ca_5_(PO_4_)_3_(OH)] incorporated in micro-cluster or nano-crystalline forms in oral care products. HA is a bioactive and biocompatible material with a chemical composition similar to the apatite crystals of human enamel.^18^, ^19^, ^20^, ^21^ A toothpaste incorporating ion-doped hydroxyapatite (Sr-Mg-CO3-HA) and semi-fluoride substituted HA (HAF) within a chitosan matrix was recently developed and introduced in toothpastes. Several *in vitro* studies have found that nano-hydroxyapatite toothpaste is able to remineralize artificial initial caries lesions.^14^, ^15^, ^16^, ^17^^,^^19^^,^^20^ The fluoride ions can interact with the hydroxyapatite structure, making it less soluble and more resistant to acidic challenges. The nano-sized particles of fluoridated hydroxyapatite can penetrate the enamel's micropores and defects, facilitating the remineralization process at a deeper level. Combining nano-sized hydroxyapatite and chitosan can promote enamel remineralization by delivering essential minerals to the tooth surface.

In 2017, a randomized clinical trial called “Enamel mineralization and caries prevention with a novel biomimetic hydroxyapatite fluoride toothpaste” was designed. The aim of the study was to evaluate the incidence of new caries lesions and/or the progression of carious lesions after regular use of a toothpaste containing biomimetic hydroxyapatite (Bioactive complex and H.A.F) and fluoride. A secondary aim of the RCT was to determine the mineral uptake released into deciduous enamel by the toothpaste.

In previous papers, HAF toothpastes were able to reduce caries increment in children over 2 years more than traditional fluoridated toothpastes.^22^^,^^23^ In particular, the trial found that toothpastes containing biomimetic HAF (1000 and 1450 ppm Fluoride), fluoride and chitosan appeared to be clinically effective in preventing new caries lesions. In contrast, fluoridated toothpaste containing sodium monofluorophosphate (NaMFP) had a slight effect only when used at the highest fluoride concentration (1450 ppmF).

Starting from this premise, a new hypothesis was formulated: HAF toothpaste might be more effective even in arresting the progression of existing lesions and enhancing the remineralization of enamel compared to standard fluoridated toothpaste. The null hypothesis of this trial is that the effect of HAF toothpaste use on carious lesion progression is similar to that obtained with NaMFP toothpastes. A triple-blind randomized clinical trial was designed and carried out to validate this hypothesis. The present study aimed to evaluate the therapeutic effect of HAF on active caries lesions compared to that of a fluoridated standard toothpaste.

## Materials and methods

### Design of the study

The planned duration of the trial was 24 months with a start date in January 2018; unfortunately, during the second year (2020) of the trial, the COVID pandemic reached Italy and, as of 9 March 2020, the government decreed a national blockade or quarantine, restricting population movements except for necessity, work and health circumstances, in response to the growing COVID-19 pandemic in the country. The lockdown also involved school activities, preventing check-ups. At the time of the lockdown, 452 (87.26%) children in the enrolled sample had completed the study. Consequently, some follow-up examinations were conducted in the winter of 2020/2021. The study was conducted at the Dental Clinic of the Sassari University, Italy. (Ethical Committee approval written in the statement) and registered at http://www.clinicaltrial.gov (NCT04906291). In the previous articles are possible to retrieve additional information about the trial.^22^^,^^23^ Since no similar studies are available, an *in vitro* study was used to calculate the sample size.^20^ As the fluoride concentration was the same in the 2 arms within each age group (HAF+F and F alone), the difference in lesion depth assessed before and after treatment with hydroxyapatite (-30.2) and with the negative control (without fluoride and hydroxyapatite) (-64.4) were used to assess the effect of hydroxyapatite on caries arrest (ES = 0.87). Power analysis was carried out with G*Power 3.1.3 for Apple, using a nonparametric Mann–Whitney U test with an effect size of 0.87^20^ and an error probability of 0.05, the number of subjects in each age group was set at 30 (60 subjects in total), with an actual power of 0.95.

The frame consisted of all the children aged 4-7 living in the study area (n = 13,239) who were considered possible participants; 64 public schools from the North-Sardinia accepted to get involved (school acceptance rate 75.60%). Children whose parents/guardians signed an informed leaflet were enrolled in the study. Classes in which consent to participate and eligibility was obtained from less than 80% of the subject were excluded for simplicity. A systematic cluster sampling procedure of randomization was conducted, considering each school class (range 14-16 children) as a cluster. Randomization was performed using Excel 2014 using a systematic cluster sampling; each school class was identified as a cluster and compiled into 2 list (1 for each age group) and a random number was assigned to each class with the random command, then classes were ordered from the lower to the higher number. To create the 4 groups (2 for each age group) to the first class the treatment was randomly chosen, while the others were selected at the systematic interval of 3 classes. Overall, 610 subjects were examined and enrolled in the trial.

For the present study, only children with at least 1 active caries lesion (see below for the definition of an active lesion) were considered eligible; further inclusion criteria were: good general health (*ie*, absence of systemic diseases), as assessed by the examiners; agreement not to use any oral hygiene products except for the toothpaste provided. Exclusion criteria were: ongoing oral or dental treatment except for emergency treatment; known allergic reaction to an oral hygiene product and/or medication and/or dental material previously used in the mouth or pharynx; allergy to any of the components of the products under investigation; participation in another clinical trial in progress or within the last 30 days; antibiotic therapy within the past 3 months.

At baseline, a standardized questionnaire was administered to parents/caregivers to gain information regarding caries risk factors as behavior habits (toothbrush frequency, use of fluoride), dietary habits (use of pacifier at night, number of meals, diet cariogenic content), socio-economic status of the children/family/caregivers, categorized according to the SocFam scale as medium-low, medium and medium-high level, and lifestyle behaviors (frequency of dental check-ups).^22^

### Treatment

All the toothpastes used in the trial were produced and supplied by Curasept S.p.A (Saronno, Italy). The toothpastes were: HAF toothpaste (1000 ppmF) containing fluoride-substituted Hydroxyapatite and magnesium-, strontium-, carbonate-substituted hydroxyapatite, in a chitosan matrix (HAF); sodium monofluorophosphate fluoridated toothpaste (1000 ppmF) without other active components (F); HAF toothpaste (1450 ppmF) and sodium monofluorophosphate fluoridated toothpaste (1450 ppmF). The treatment allocation was concealed; all tubes were identical in weight and shape and were marked by colour (white or green) according to the group. The code was sealed and was only disclosed at the end of the trial.

The children were instructed to brush their teeth with a manual toothbrush for at least 2 minutes after each main meal (3 times/day), once at school under the supervision of a teacher and twice at home. The compliance and any observed side effects of the products were measured by means of a questionnaire administered to the participants’ parents each month for the duration of the trial as previously reported.^22^ In the same questionnaire was also investigated the possible use of other fluoridated products (ie, salt, gels, mouthwashes) that were not allowed throughout the trial. To evaluate the success of the trial, participants were given toothpaste necessary for 2 months at a time and asked to return the empty pack when receiving the new 1 for the following months. During the lockdown, 1 of the authors (CS) contacted all the children and the families 3 times per week via Zoom to check the correct use of the toothpastes. During the trial, parents could follow their pediatric dentist's recommendations for treating carious lesions in their children. No further preventive treatment (*ie,* topical application of fluoride or sealants) was allowed.

### Caries activity evaluation

The calibration of examiners (4) was performed comparable with the trial conditions, prior of the start of the trial and repeated before the interim examination (12 months) and before the last examination (24 months). An author acted as benchmark examiner (GC); at baseline the training comprised: 1- a theoretical course (6 h) with a guidance manual; 2- examination of extracted teeth (20 primary/20 permanent) plus a session of 100 photographs of extracted teeth; 3- a clinical training involving the examination of 60 children with a wide range of clinical caries situation. The subjects were re-examined after 72hrs. Inter-and-intra-examiner reliability was evaluated: the first comparing the benchmark (GC) and the examiners outcomes through fixed-effect analysis of variance; the second as the percentage of agreement using Cohen's kappa statistic.^22^ A good inter-examiner agreement was recorded for sound teeth *P* = .22, mean square of error = 0.51 F = 1.45; for activity of no-cavitated caries lesions *P* = .32, mean square of error = 0.51 F = 1,42; for activity of cavitated caries lesions *P* = .21 mean square of error = 0.46 F = 1.44. Intra-examiner reliability was also quite high (Cohen's kappa = 0.91, 0.84 and 0.85 for sound teeth, noncavitated lesions, cavitated lesions, respectively). Before the interim and the last follow-up examination, calibration was also run and achieved; 30 children not being enrolled into the clinical trial were examined and re-examined after 72hrs (inter-examiners reliability mean square of error = 0.46 *P* = .24 F = 1.67 and intra-examiner reliability Cohen's kappa = 0.89). Before the last examination, in view of the high values of inter and intra-examiner reliability, only 20 children not enrolled into the trial were examined and re-examined, and the inter- and intra-examiner reliability values found previously were confirmed. Caries activity was recorded at baseline and at follow-up at more than 24 months. The children were seated on a chair, while the examiner was behind them wearing a head light; the tooth surfaces were cleaned and dried with gauze and examined with a mirror and a periodontal probe. Activity status was defined by clinical surface features.^24^ In enamel, a caries lesion was registered if a change in texture, translucency, and color (yellow, brown, black) was observed; if all 3 characteristics were altered, the lesion was classified as “active”; if all 3 characteristics were not altered, the lesion was classified as “inactive”; if 1 or 2 characteristics were altered, the lesion was defined as “partially active.” In dentine, a caries lesion was registered if characteristics of lesion classified as 4 or 5 according to ICDAS were observed; the caries activity was categorized as “inactive,” if the lesion was hard and smooth when the tip explorer was moved gently across its surface; as “firm/leathery,” when the sensation of the probe against the lesion was physically resistant; as “active” if the texture was soft.^4^^,^^8^, ^9^, ^10^, ^11^ The caries activity was assessed only via the visual-tactile examination as radiography was infeasible and impractical in children.

### Data analysis

Only data from children who completed the entire follow-up period were included in the analysis. To ensure anonymity and to monitor changes of the caries lesion during the study, an alphanumeric code was assigned per community/municipality, school, and class, and within each class, subjects were coded in alphabetical order and, therefore, outcomes blinded to the participants' group assignments. The unit of analysis was each affected tooth (primary and permanent). Study data were collected and managed using REDCap electronic data capture tools hosted at the University of Bern (Switzerland).^25^ “REDCap (Research Electronic Data Capture) is a secure, web-based software platform designed to support data capture for research studies, providing (1) an intuitive interface for validated data capture; (2) audit trails for tracking data manipulation and export procedures; (3) automated export procedures for seamless data downloads to common statistical packages; and (4) procedures for data integration and interoperability with external sources.”^25^ All collected data were entered into an ad hoc spreadsheet (Microsoft Excel 2021 for Mac, Version 16.4.8, Redmond, Washington, USA). Statistical analyses were performed using Stata/SE1 software (StataCorp LLC, College Station, Texas, USA), version Stata/SE® 17 for Mac (Intel 64-bit).

The primary outcome was the change in lesion status at the end of the treatment protocol. A per-protocol analysis was adopted, thus excluding children lost to follow-up. Chi-squared or Fisher's exact tests compared the groups for lesion count variables. For the primary outcome the proportion of change of status at 24 months of follow-up (intention to treat analysis using multiple imputation^26^^,^^27^ also considering baseline variables was per- formed as a sensitivity analysis using superiority test P value and confidence interval (CI = 95%).^27^ The significance level was set at 5% for all tests.

## Results

Of the potential participants aged 4-7 years living in the study area (n = 13,239), 610 children were enrolled. In fact, 78.22% of the potential population (n = 10,356) did not consent to participation either as a whole school or as individual parents (4022 subjects aged 4-5 years; 6334 subjects aged 6-7 years). Of the 28,833 potentially eligible subjects, 78.8447% did not fulfil the inclusion criteria (758 subjects aged 4-5 years; 1515 subjects aged 6-7 years).

**Fig. 1:**
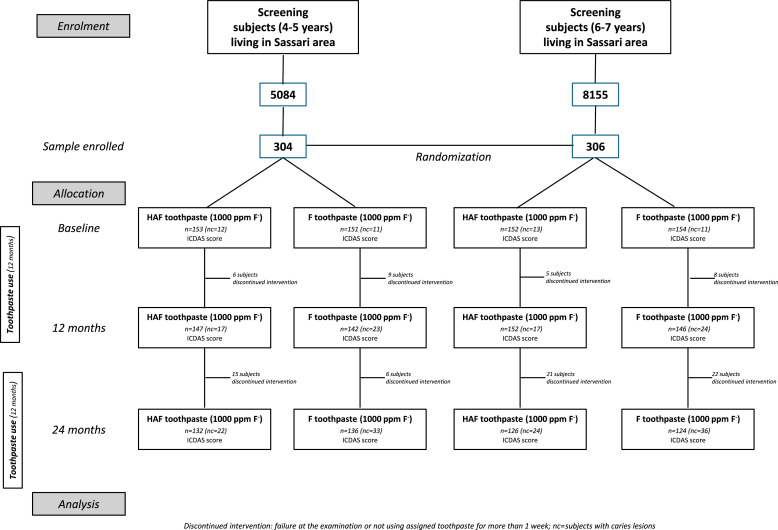
CONSORT flow chart of the overall trial.^23^

As mentioned above, this study reports only data from children who completed the trial following a per-protocol approach, 518 children in total; Intention to Treat analysis was also performed and results are presented in Supplemental file. The demographic characteristics of the sample enrolled is presented in supplemental materials (Table 1s). Below it is displayed the overall Consort flow of the Randomized Clinical Trial (Figure 1). The dropout rate was 15.08%, with restriction due to Covid-19, family moving to another area or family/child deciding to leave the trial, as the main dropout reasons. The number of subjects with caries lesions at each time point is also shown in Figure 1.

**Table 1:** Caries lesions status in enamel and dentine in primary and permanent dentition during the trial. χ^2^ test with Yates correction for continuity was calculated to assess difference between the 2 groups, in case a cell has a count less than 5 the Fisher exact test was used.

**Primary teeth**							
***Enamel***							
	*Baseline*	*Two-years follow-up*					
		*Change of status*					
	*lesions*	*progressed*	*reversed*	filled*	*exfoliated*	*new lesions*	*Total lesions*
HAF_(1000)_	45	1	1	7	2	14	28
HAF_(1450)_	134	3	2	10	3	16	149
HAF	*179*	*4*	*3*	*17*	*5*	*30*	*187*
NaMFP_(1000)_	44	3	2	8	4	23	95
NaMFP_(1450)_	130	4	2	11	5	35	109
NaMFP	174	7	4	19	9	58	204

			*Fisher exact test =7.15 p<0.01*				


***Dentine***							
	*Baseline*	*Two-Years follow-up*					
		*Change of status*					
	*lesions*	filled*	*exfoliated*	*New lesions*	*Total lesions*		
HAF_(1000)_	42	27	10	17	41		
HAF_(1450)_	197	55	13	29	139		
HAF	239	82	23	46	180		
NaMFP_(1000)_	45	24	6	26	54		
NaMFP_(1450)_	192	60	13	47	153		
NaMFP	237	84	19	73	207		

			*χ^2^ Yates =4.77 p=0.03*				

**Permanent teeth**							
***Enamel***							
	*Baseline*	*Two-Years follow-up*					
		*Change of status*	*New lesions*	*Total*			
	*Lesions*	*progressed/reversed*	*Lesions*	*Lesions*			
HAF	47	19	12	40			
NaMFP	60	25	21	47			

*χ^2^ Yates =0.32* P *= .57*							

*Dentine*							
	*Baseline*	*Two-Years follow-up*					
	*lesions*	Change of status filled*	*New lesions*	*Total*			
			*lesions*	*lesions*			
HAF	29	18	22	40			
NaMFP	26	17	28	47			

		*χ^2^ Yates = 0.33* P *= .56*					

⁎ progressed lesion requiring filling as treatment.

In primary teeth, at baseline, 3.28% of the teeth had caries in enamel and 4.39% in dentine in the HAF group, while in the NaMFP group, 3.22% and 4.38% of the teeth presented caries lesions in enamel and dentine, respectively (*P* = .89). In permanent dentition, at the baseline, in the HAF group, 4.17% of teeth had enamel caries and 2.57% dentinal caries. In comparison, in the NaMFP group, 3.54% and 4.13% of the teeth had caries lesions in enamel and dentine, respectively (Table 1).

**Fig. 2:**
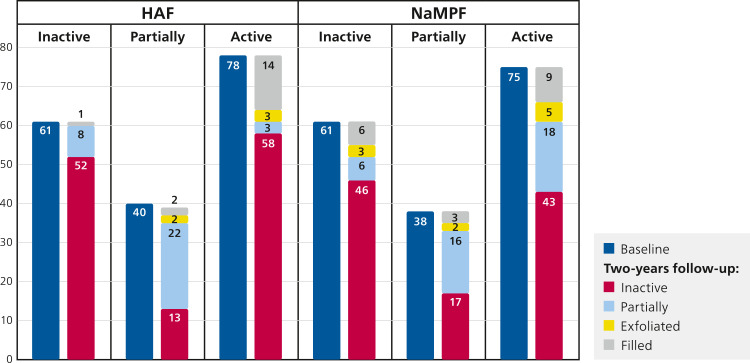
Enamel lesions activity in primary dentition at baseline and at the 2-year follow-up in the 2 groups.

**Fig. 3:**
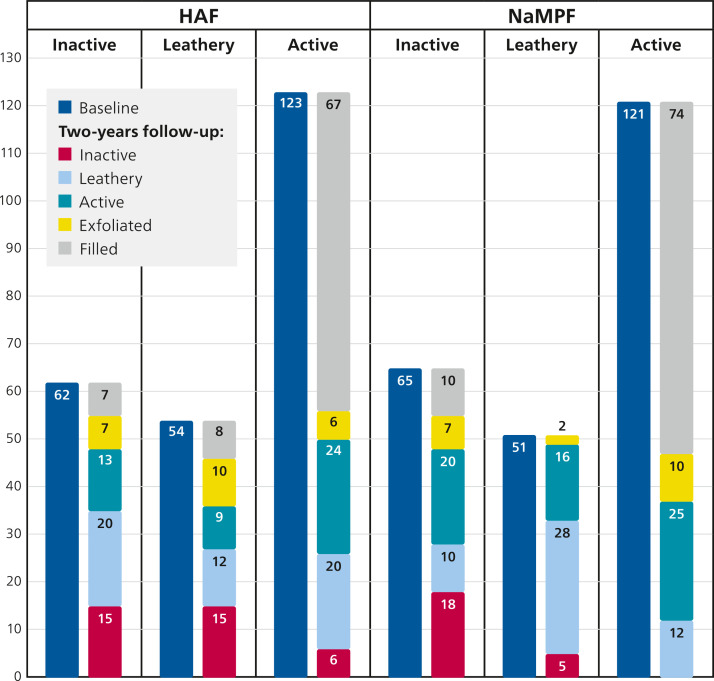
Dentinal lesions activity in primary dentition at baseline and at the 2-year follow-up in the 2 groups.

Activity status during the study period was only assessed for primary teeth (Table 2; Fig. 2, Fig. 3). At baseline, there was no statistically significant difference between the 2 groups (HAF and NaMFP) in enamel and dentinal lesions (*P* = .98 and *P* = .92, respectively). At the final examination, a higher number of lesions, both in enamel and dentin, were observed in the NaMFP group compared to the HAF toothpaste group. However, the difference was statistically significant only for enamel lesions (*P* < .01).

**Table 2:** Activity status of caries lesions in primary dentition. Only self-cleansing lesions were considered. χ^2^ test with Yates correction for continuity was calculated to assess difference between the 2 groups, in case a cell has a count less than 5 the Fisher exact test was used.

Baseline										
*-Enamel*										
	*Inactive*		*Partial Active*		*Active*		*Total*			
	*N*	*%*	*N*	*%*	*N*	*%*	*N*			
HAF	61	34.08	40	22.35	78	43.57	179			
NaMFP	61	35.06	38	21.84	75	43.10	174			
*χ^2^ Yates = 0.04 P = .98*										

*-Dentine*										
	*Inactive*		*Leathery*		*Active*		*Total*			
	*N*	*%*	*N*	*%*	*N*	*%*	*N*			

HAF	62	25.94	54	22.60	123	51.46	239			
NaMFP	65	27.43	51	21.52	121	51.05	237			
*χ^2^ Yates = 0.16 P = .92*										

Two-Year follow-up										
*-Enamel*										
	*Inactive*		*Partial Active*		*Still Active*		*Active (new lesions)*		*Total*	*Filled teeth*
	*N*	*%*	*N*	*%*	*N*	*%*	*N*	*%*	*N*	*N*

HAF	123	65.78	28	14.97	–	–	30	16.04	181	17
NaMFP	116	56.86	40	19.61	–	–	58	28.43	214	19
*χ^2^ Yates = 8.53 P < .01*										

*-Dentine*										
	*Inactive*		*Leathery*		*Still Active*		*New Lesions*		*Total*	*Filled teeth*
	*N*	*%*	*N*	*%*	*N*	*%*	*N*	*%*	*N*	*N*

HAF	52	28.89	58	32.22	24	13.33	46	25.56	180	82
NaMFP	48	23.19	61	29.47	25	13.37	73	35.27	207	84
*χ^2^ Yates = 5.13 P = .08*

In the HAF group, 61 enamel lesions were inactive at baseline, of which only 1 was filled at the end of the study. Of the 40 partially active lesions at baseline, 13 were inactive at the 2-year follow-up. Of the active lesions (n = 78) at baseline, nearly 3-quarters (n = 58) were inactive at the follow-up. In the NaMFP group, only 3 lesions coded as inactive at the start of the study were coded as filled at the end of the trial; half of the lesions were coded as inactive. The difference between the 2 groups regarding the change of activity of the lesion (active at baseline and inactive at follow-up) was statistically significant (χ^2^ = 4.21 *P* = .04).

In the dentine, 8 (12.90%) lesions in the HAF group, inactive at baseline, were filled at the 2-year examination. A similar percentage (15.38%, n = 10) was observed in the group treated with NaMFP. In the HAF group, 20% (n = 24) of the lesions recorded as active at baseline were still active at follow-up; moreover, 67 active lesions at baseline were recorded as filled at the 2-year evaluation. Similar figures were observed in the MPF group: 20% (n = 25) of the lesions recorded as active at baseline were still active at follow-up. In the HAF group, 15 lesions (27.77%) were inactive at follow-up, while less than 10% (n = 5) of lesions at baseline in the NaMFP group were inactive at follow-up; the difference between groups was statistically significant (χ^2^ = 4.39 *P* = .03). The Intention to treat analysis (ITT) can be found in the supplemental materials (Table 2). The rates between number of lesions recorded at 24 months-follow-up and the number of teeth examined at baseline were 6.73% and 7.61% for HAF and MPF analysis groups respectively in the primary dentition (*P* = .04); in the permanent dentition the rates were 13.8 and 15.82, respectively (*P* = .03).

## Discussion

The research project was designed to test the hypothesis that HAF toothpaste with fluoride might be more effective even in arresting the progression of existing lesions and enhancing the remineralization of enamel compared to standard fluoridated toothpaste.

Based on this concept, it was decided to consider together the 2 HAF toothpastes compared to the 2 fluoridated (F) toothpastes.

This trial proved that both fluoridated toothpastes tested could arrest caries' progression and remineralize carious lesions. The caries activity was assessed via the visual-tactile examination as radiography was infeasible and impractical in children.^24^ For clarity, the outcome was more marked in the HAF group than in the NaMFP group. Thus, the outcomes validate the hypothesis, in agreement with the previous findings of this RCT, in which a HAF toothpaste showed to produce a reduced caries increment in children over 2 years more than traditional fluoridated toothpastes.^22^^,^^23^

Initially, the ITT analysis was not planned in the RCT, as the present study is quite peculiar as include kindergarten and school children that during the trial entered in mixing dentition phase. Since the tooth and not the subject is the unit of analysis, the mixed dentition phase creates problems with regard to ITT, because it is not possible to assess the number of teeth present in subjects who left the trial.

The calculation of the incidence is also not simple because it is not merely counting new lesions but assessing how many of these lesions have progressed or have been filled and are therefore no longer lesions and especially how many teeth with lesions are exfoliated during the trial (gross vs net incidence). The ITT is, nevertheless, commonly proposed in the analysis strategy for the reporting of clinical trials as it involves non-compliant participants in the trial, thereby increasing the external validity of the results.^26^^,^^27^ In the present trial, an ITT analysis using multiple imputations was performed confirming the results more pronounced in the HAF group than in the NaMFP group.

It is well known that dental caries is a preventable disease,^28^, ^29^, ^30^ but its prevalence is still high.^2^^,^^31^ The new philosophy in the management of carious disease is the nonoperative treatment; it is therefore necessary to move to a new concept of a nonsurgical but medical model.^9^ Fluoride remains the essential key to this approach.^31^ The World Health Organization passed a resolution stating that universal access to fluoride for caries prevention is a basic human right.^31^ Water fluoridation is an integral part of oral health programs worldwide.^32^^,^^33^

The primary outcome measure of the present trial was rather unusual, halting the progression of caries lesions and children moving from a caries-free to a caries-active state.

The choice of the trial primary outcome measure fits perfectly into the policy context of the draft global oral health action plan (2023-2030) of the World Health Organization.^32^ During the design and planning of the study, the WHO (World Health Organization) Model List of Essential Medicines (EML) was updated to include a new section on "dental preparations," adding fluoride toothpaste, silver diamine fluoride (SDF) and glass ionomer cement.^32^^,^^33^

In Western countries, the need to reassess the policy of a state-funded system of universal prevention in children is being debated given the considerable costs involved in providing this service. In Italy, the only method for caries prevention available for most children is through oral hygiene maintenance.^2^^,^^34^^,^^35^ The main effect on caries lesions in dentine is the activity change that depends on the progression depth and surface involved.^36^ The present trial uses products that cannot be compared with studies using only fluoride derivatives that have shown greater efficacy in arresting dentinal carious lesions.^14^ It is possible to hypothesize that the presence of HA facilitates the remineralization of initial enamel lesions to a greater extent.

There were some limitations to the trial that need to be addressed. One potential limitation is the possible occurrence of cross-level bias. By randomly assigning participants to different treatment groups, randomization made each group comparable at baseline in terms of observed and unobserved characteristics. This comparability reduces the likelihood that differences in outcomes between groups are due to factors other than the treatment itself.^37^ In this study, randomization was essential to control for potential confounding factors and ensure that observed differences in caries progression and enamel remineralization could be attributed to the type of toothpaste used, rather than other variables such as socioeconomic status, baseline oral health or behavior. Thus, randomization not only strengthens the validity of the results, but also mitigates the risk of cross-sectional bias that could distort the conclusions on the efficacy of HAF toothpaste. The enrolment phase and consequent follow-up examinations of the participants took place over an extended period, and the risk of selection bias might also be considered. The study duration was also complicated due to the different waves of the COVID-19 pandemic that slowed down the follow-up examinations for several months. The caries activity scoring system must be considered reliable as the intra-examiner tests and inter-examiner calibration showed high agreement values, even the examinations were carried out in a school setting not in a dental chair. The scientific evidence for compliance with professional varnish treatment is high, whereas compliance with recommended home oral care is still a concern.^38^ No problems were reported by participants/parents/caregivers regarding the toothpaste used; moreover, the costs of trials, including professional fluoride, are relatively cost-effective, especially in preschool children.^39^^,^^40^ Another shortcoming may be that a subgroup analysis of fluoride concentration or health economic evaluation was not carried out. Otherwise, the study's target population was relatively homogeneous regarding socioeconomic background. Furthermore, no adverse effects were reported by children or ascertained clinically. Previous clinical studies observed similar findings and reported no safety issues with nano-hydroxyapatite in oral care products.^41^^,^^42^

The HAF group toothpaste could provide several benefits for enamel remineralization. Nano-fluoridated hydroxyapatite can attract and adsorb calcium and phosphate ions from saliva or fluoride-containing dental products. These ions contribute to forming new mineral crystals, helping replenish lost minerals in the enamel. Nano-fluoridated hydroxyapatite can enhance the acid resistance of tooth enamel. However, it's important to note that further research and clinical studies are needed to understand this technology's efficacy and long-term effects.

The findings of this study underscore the significant potential for using HAF toothpaste in community settings for the prevention and management of dental caries in children. The demonstrated efficacy of HAF toothpaste in not only arresting the progression of existing carious lesions but also enhancing the remineralization of enamel suggests it could be a powerful tool in public oral health initiatives.

Given the high prevalence of dental caries and the associated costs of traditional restorative treatments, integrating HAF toothpaste into routine dental care protocols could offer a cost-effective, non-invasive solution. This aligns with the contemporary shift towards a medical model of caries management, emphasizing prevention and non-operative treatment over surgical interventions. Public health policies should consider incorporating HAF toothpaste as part of broader caries prevention programs, particularly in settings where access to dental care is limited.

However, to maximize the public health impact of this approach, further research is needed. Future studies should focus on determining the optimal dosage and frequency of HAF toothpaste use to ensure maximum efficacy across diverse populations. Additionally, feasibility testing in various settings and scenarios is essential to address generalizability.

## Conclusions

Based on the above outcome, it is possible to speculate that HAF-containing toothpaste may be a better choice for children with active caries lesions in primary dentition than F-containing toothpaste. Moreover, the use of HAF in oral care products may have different dosages for infants, children, and adults, even if the efficacy needs to be confirmed through further clinical trials.

## Author's contribution

F.C. and C.S. Contributed to conception, review the literature, design, data acquisition, statistical analysis and interpretation, critically revised the manuscript. RJW. Contributed to conception, design, drafted and critically revised & editing the manuscript. TGW. Contributed to conception, review the literature drafted and criti-cally revised & editing the manuscript. A.A. Contributed to conception, statistical interpretation, manuscript writing, critically revised the manuscript. M.G.C. Contributed to conception, design, drafted and critically revised the manuscript, study design, manuscript writing, review final re-vision of the manuscript. G.C. Contributed to conception, design, formal analysis, methodology, patient qualification for the study, data and statistical analysis, drafted and critically revised the manuscript.

## Institutional review board statement

The study was approved by the Ethics Committee of the University of Sassari (Ethical Committee approval n 217/2017 Sassari) and registered at http://www.clinicaltrial.gov. (NCT04906291). All performed procedures were in accordance with the ethical standards of the 1964 Helsinki Declaration and its later amendments.

## Informed consent statement

Informed consent was obtained from all subjects involved in the study. A letter explaining the purpose of the study and the informed consent were distributed to the parents/caregivers of children of the 2 age groups considered. A total of 610 subjects ful-filling the criteria of inclusion accepted to participate.

## Data availability statement

The dataset generated during and/or analyzed during the current study are not publicly available due to data holder restrictions. The data are available on request.

## Conflict of interests

None disclosed.

## Appendix Supplementary materials

Image, application 1

## Footnotes

Supplementary material associated with this article can be found in the online version at doi:10.1016/j.identj.2024.09.037.
