# Supplementary material for: Hydroxyapatite-Fluoride Toothpastes on Caries Activity: A Triple-Blind Randomized Clinical Trial
Source: Int Dent J. 2025 Feb 18;75(2):632–42. doi: 10.1016/j.identj.2024.09.037 (PMC11976554; doi:10.1016/j.identj.2024.09.037)
Supplement: Supplementary file 1 [file mmc1.docx]

**Table 1S.** Oral Hygiene habits, dietary behaviors, socio-economic status and dental check-up frequency of the children enrolled in the trial in the two groups [23].

|  | 4-5 years  *n (%)* | |  | 6-7 years  *n (%)* | |
| --- | --- | --- | --- | --- | --- |
|  | *HAF_1000_* | *F_1000_* |  | *HAF_1450_* | *F_1450_* |
| *Toothbrush frequency* | | | | | |
| *<2 a day* | 68 (22.37) | 72 (23.68) |  | 26 (8.50) | 28 (9.15) |
| *>2 a day* | 85 (27.96) | 79 (25.99) |  | 126 (41.12) | 126 (41.12) |
| *Use of fluoridated toothpaste* | | | | | |
| *No* | 44 (14.47) | 42 (13.81) |  | 13 (4.25) | 14 (4.58) |
| *Yes* | 109 (35.86) | 109 (35.86) |  | 139 (45.42) | 140 (45.75) |
| *Use of pacifier at night* | | | | | |
| *No* | 64 (21.05) | 61 (20.07) |  | 145 (47.39) | 143 (46.73) |
| *Yes* | 89 (29.28) | 90 (29.60) |  | 7 (2.29) | 11 (3.59) |
| *Main meals frequency* | | | | | |
| *2 a day* | 7 (2.30) | 8 (2.63) |  | 15 (4.90) | 12 (3.92) |
| *3 a day* | 42 (13.82) | 39 (12.83) |  | 130 (42.48) | 135 (44.12) |
| *>3 a day* | 104 (34.21) | 104 (34.21) |  | 7 (2.29) | 7 (2.29) |
| *Cariogenic diet* | | | | | |
| *No* | 7 (2.30) | 7 (2.30) |  | 19 (6.21) | 23 (7.52) |
| *Yes* | 146 (48.03) | 144 (47.37) |  | 133 (42.48) | 131 (44.12) |
| *Socio-economic status* | | | | | |
| *Medium-Low* | 87 (28.62) | 81 (26.64) |  | 66 (21.57) | 67 (21.90) |
| *Medium* | 48 (15.79) | 44 (14.47) |  | 63 (20.59) | 57 (18.63) |
| *Medium-High* | 18 (5.93) | 26 (8.55) |  | 23 (7.52) | 30 (9.80) |
| *Dental check-ups frequency (parents/caregivers)* | | | | | |
| *In case of problem* | 96 (31.58) | 95 (31.25) |  | 93 (30.39) | 87 (28.43) |
| *Rarely* | 40 (13.16) | 36 (11.84) |  | 41 (13.40) | 43 (14.05) |
| *Routinely* | 17 (5.60) | 20 (6.58) |  | 22 (7.19) | 20 (6.54) |

**Table 2S**. Intention to treat analysis (24 months) as the difference of lesions in the two groups.

|  | **HAF toothpaste** | **MFP toothpaste** | *p-value* |
| --- | --- | --- | --- |
| *Primary dentition* | | | |
| n of lesions/n of teeth | 367/5446 | 411/5446 |  |
| Incidence (%) | 6.73 | 7.61 | 0.04 |
| Absolute difference (_95%_CI) | 0.88 (1.02 – 1.36) | |  |
| Relative Risk RR (_95%_CI) | 0.78 (0.66 – 0.91) | | 0.04 |
|  |  |  |  |
| *Permanent dentition* | | | |
| n of lesions /n of teeth | 156/1128 | 180/1138 |  |
| Incidence (%) | 13.83 | 15.82 | 0.03 |
| Absolute difference (_95%_CI) | 1.99 (1.73 – 2.44) | |  |
| Relative Risk RR (_95%_CI) | 0.71 (0.54 – 0.88) | | 0.04 |
